# North vs south differences in acute peptic ulcer hemorrhage in Croatia: hospitalization incidence trends, clinical features, and 30-day case fatality

**DOI:** 10.3325/cmj.2014.55.647

**Published:** 2014-12

**Authors:** Neven Ljubičić, Tajana Pavić, Ivan Budimir, Željko Puljiz, Alen Bišćanin, Andre Bratanić, Marko Nikolić, Davor Hrabar, Branko Troskot

**Affiliations:** 1 Division of Gastroenterology, Department of Internal Medicine, “Sestre Milosrdnice” Clinical Hospital Center, University of Zagreb School of Medicine and School of Dental Medicine, Zagreb, Croatia; 2 Division of Gastroenterology, Department of Internal Medicine, Clinical Hospital Center Split, University of Split School of Medicine, Split, Croatia

## Abstract

**Aim:**

To assess the seven-year trends of hospitalization incidence due to acute peptic ulcer hemorrhage (APUH) and associated risk factors, and examine the differences in these trends between two regions in Croatia.

**Methods:**

The study collected sociodemographic, clinical, and endoscopic data on 2204 patients with endoscopically confirmed APUH who were admitted to the Clinical Hospital Center “Sestre Milosrdnice,” Zagreb and Clinical Hospital Center Split between January 1, 2005 and December 31, 2011. We determined hospitalization incidence rates, 30-day case fatality rate, clinical outcomes, and incidence-associated factors.

**Results:**

No differences were observed in APUH hospitalization incidence rates between the regions. Age-standardized one-year cumulative APUH hospitalization incidence rate calculated using the European Standard Population was significantly higher in Zagreb than in Split region (43.2/100 000 vs 29.2/100,000). A significantly higher APUH hospitalization incidence rates were observed in the above 65 years age group. Overall 30-day case fatality rate was 4.9%.

**Conclusion:**

The hospitalization incidence of APUH in two populations did not change over the observational period and it was significantly higher in the Zagreb region. The incidence of acute duodenal ulcer hemorrhage also remained unchanged, whereas the incidence of acute gastric ulcer hemorrhage increased. The results of this study allow us to monitor epidemiological indicators of APUH and compare data with other countries.

Acute peptic ulcer hemorrhage (APUH) is a common medical condition that results in high patient morbidity. In spite of pharmacological and endoscopic therapy advancements, APUH remains a significant medical problem due to still unresolved questions regarding acute management and complications (1). Mortality rates from ulcer hemorrhage in various European countries range from 3.4% to 14%, and the reason for this difference is unknown (1-3). While the prevalence of *H. pylori* infection declines, there is an increase in the proportion of patients with ulcer hemorrhage unrelated to *H. pylori* infection or the use of non-steroidal anti-inflammatory drugs (NSAIDs), including aspirin (4). Recent data suggest that patients with non-*H. pylori*-non-NSAID idiopathic bleeding peptic ulcers have significantly higher mortality rates than patients with *H. pylori* ulcers (5). Numerous studies have shown that endoscopic and pharmacological treatment of APUH episodes can reduce rebleeding rates, rates of surgical interventions, and length of hospital stay, although some trials indicate that none of these approaches has substantially reduced the overall mortality associated with bleeding events (3,4,6). So far no data has been published on the hospitalization incidence of APUH in Croatia. The aim of this study was to assess the seven-year trends in hospitalization incidence due to APUH, clinical features of APUH, and 30-day case fatality in two different regions in Croatia.

## Methods

This study was conducted in two centers that together serve a population of approximately 760 000: Clinical Hospital Center “Sestre Milosrdnice” in Zagreb – northern region of Croatia, serving a population of 300 000 people and Clinical Hospital Center Split – southern region of Croatia, serving a population of 460 000 people. 4571 consecutive patients admitted to these centers with acute upper gastrointestinal hemorrhage between January 1, 2005 and December 31, 2011 were considered for inclusion. Only the first admission of each patient was counted, irrespective of subsequent or previous episodes of upper gastrointestinal hemorrhage. Acute upper gastrointestinal hemorrhage was suspected if the patient presented with hematemesis, melena, or hematochezia and/or if bloody nasogastric aspirate was observed. In all such patients, upper gastrointestinal endoscopy was performed within 12 hours of hospital admission. Patients were included in the study only if emergency endoscopy revealed a gastric or duodenal ulcer without any other source of bleeding. The following patients’ data were collected: sex, age, smoking and alcohol consumption, recent use of NSAIDs and/or antiplatelet agents (aspirin, clopidogrel, and ticlopidine), medical history, and physical and initial laboratory findings (complete blood count, prothrombin time, and renal function tests). Drug usage was defined as at least one dose taken within one week before the episode of hemorrhage. The overall health and comorbidity was graded according to the American Society of Anesthesiology (ASA) classification (7). The hemodynamic instability was defined as syncope or systolic blood pressure <100 mm Hg. Risk assessment of APUH was performed according to the Rockall classification (8).

Endoscopy was carried out by trained endoscopists, each with at least five years of experience in treating patients with gastrointestinal (GI) hemorrhage. Endoscopic characteristics including ulcer localization, ulcer size, and signs of hemorrhage were recorded. The diameter of ulcer was measured using endoscopic biopsy forceps (FG-25K, Olympus, Tokyo, Japan). Hemorrhage was classified according to the modified Forrest criteria (9). The commonly used hemostatic procedures were epinephrine injection (1:10 000 solution of epinephrine) and/or mechanical hemostasis with stainless steel hemoclips (Olympus). All patients were given acid suppressive therapy: pantoprazole 80 mg i.v. (bolus) and then 40 mg i.v. every 8 hours for at least 48 hours, followed by 40 mg daily orally, or esomeprazole 80 mg i.v. (bolus), and then 40 mg 15 i.v. every 8 hours for at least 48 hours, followed by 40 mg daily orally. Primary hemostasis was defined as absence of hemorrhage immediately after the initial endoscopic hemostasis, whereas permanent hemostasis was defined as the absence of recurrent bleeding within the 30-day period after the initial or secondary endoscopic hemostasis. Recurrent bleeding was defined as one or more signs of ongoing hemorrhage, including fresh hematemesis or melena, hematochezia, aspiration of fresh blood via nasogastric tube, and hemoglobin level decrease by more than 2 g/dL over a 24-hour period. If recurrent hemorrhage was suspected, upper endoscopy was performed immediately. Recurrent hemorrhage was confirmed in case of active bleeding, presence of fresh blood clot in the ulcer base or “coffee ground” material or blood in the stomach and/or duodenum. Patients with two unsuccessful endoscopic retreatments underwent emergency surgery.

Gastric biopsies for *H. pylori* diagnosis were performed in 1932 patients (87.6%) during index endoscopy. Two biopsy specimens were taken from the gastric antrum and body, and the presence of *H. pylori* was assessed by histologic examination of the specimens using hematoxylin-eosin (HE) stain. In patients with gastric ulcer without recurrent bleeding, control endoscopy was performed 4-5 days after the initial hemostasis, and biopsy specimens were obtained from the margins and the base of gastric ulcers to exclude malignancy. Patients with suspected or proven malignancy (n = 56) were not included in the study. In both gastric and duodenal ulcer patients with negative *H. pylori* histology at index endoscopy, urea-breath test or control endoscopy with histology was performed following cessation of acid suppressive therapy for at least 2 weeks.

Clinical outcome was recorded for up to 30 days and was defined as the amount of red blood cell transfusion, need for surgical intervention, days of hospitalization, and 30-day mortality. Written informed consent was obtained before endoscopy and enrollment. Ethical approval was obtained from ethics committees of both hospitals.

### Statistical analysis

All analyses were performed with the statistical package STATISTICA, version 10, (*www.statsoft.com*). The total population of Zagreb and Split covered by the two studied hospitals according to data from 2005 was used as the standard. Crude age-specific rates were determined for each region for both sexes and for total sample. Direct standardization using European standard population from 2013 was performed for all crude rates, and adequate rate ratios were determined. Distribution of the European Standard Population for our study was 67 000 for age group 15-65 years, 9000 for age group 65-79 years, and 2000 for age group older than 80 years (10). Confidence intervals (CI) were used to present differences between the groups The age-standardized rate for a particular condition (gastric or duodenal ulcer bleeding) was obtained by applying the observed age-specific rates for the condition to a given standard population.

## Results

**Figure 1:**
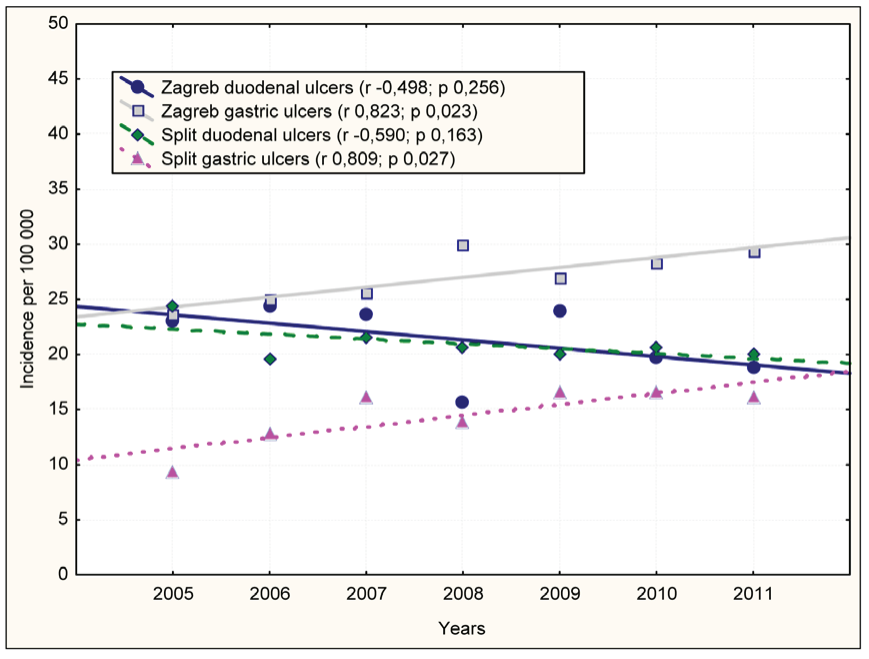
Hospitalization incidence of gastric and duodenal ulcer hemorrhage during the seven-year period (2005-2011) in two regions in Croatia

Data on 2204 patients with endoscopy-confirmed APUH were analyzed, yielding a one-year cumulative age-standardized hospitalization incidence of 41.4 cases/100 000 people (95% CI, 39.7-43.2). Age-standardized one-year cumulative APUH hospitalization incidence rate calculated using the European Standard Population in Zagreb region was 43.2/100 000 (95% CI, 40.6-45.8) and it was significantly higher than in Split region (29.2/100 000 [95% CI, 27.5-30.9]) (Table 1). The incidence of acute duodenal ulcer hemorrhage remained unchanged during the study period, whereas the incidence for acute gastric ulcer hemorrhage increased (Figure 1).

**Table 1:** Age-standardized hospitalization incidence rates per 100 000 population for gastric and duodenal peptic ulcers hemorrhage using the European Standard Population (9)

Groups	Zagreb				Split			
	participants in this study*	population*	age-specific	age -standardized	participants in this study*	population*	age-specific	age-standardized
Men								
<65 years, n	368	781,747	47	315,3963	365	1,260,909	29	1,939,473
65-80 years, n	237	144,627	164	1,474,825	302	230,284	131	1,180,282
≥80 years, n	67	31,856	210	420,643	99	54,174	183	365,491
Overall crude incidence (95% confidence interval)	70.1 (64.8-75.4)				49.6 (46.1-53.1)			
Overall age standardized incidence^†^	64.7 (59.8-69.6)				44.7 (41.5-47.8)			
Rate ratio Zagreb vs Split	1.41							
Rate ratio Zagreb vs Split standardized	1.45							
Women								
<65 years, n	127	864,603	15	984,151	69	1,269,573	5	364,138
65-80 years, n	164	205,975	80	716,590	155	296,055	52	471,197
≥80 years, n	100	71,191	140	280,933	121	109,005	111	222,007
Overall crude incidence (95% confidence interval)	34.2 (30.9-37.6)				20.6 (18.4-22.8)			
Overall age standardized incidence (95% confidence interval)^†^	25.4 (22.9-27.9)				13.6 (12.1-15.0)			
Rate ratio Zagreb vs Split	1.66							
Rate ratio Zagreb vs Split standardized	1.87							
Total								
<65 years, n	495	1,646,343	30	2,014,464	464	2,530,482	18	1,228,540
65-80 years, n	401	350,606	114	1,029,361	457	526,339	87	781,436
≥80 years, n	167	103,050	162	324,115	220	163,179	135	269,642
Overall crude incidence (95% confidence interval)	50.6 (47.6-53.7)				35.4 (33.4-37.5)			
Overall age standardized incidence (95% confidence interval) ^†^	43.2 (40.6-45.8)				29.2 (27.5-30.9)			
Rate ratio Zagreb vs Split	1.42							
Rate ratio Zagreb vs Split standardized	1.47							

*Data from 2005-2011.

†Age-standardized rates per 100 000 population for specific age groups calculated using the European Standard Population. All differences were significant (*P* < 0.05) in favor of the Zagreb region.

**Figure 2:**
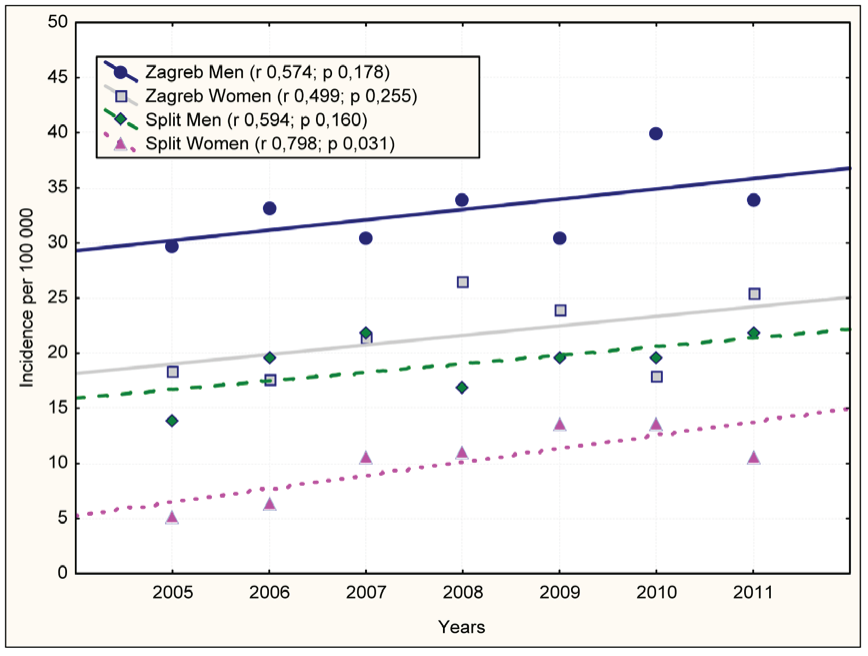
Hospitalization incidence of gastric ulcer hemorrhage in men and women during the seven-year period (2005-2011) in two regions in Croatia

**Figure 3:**
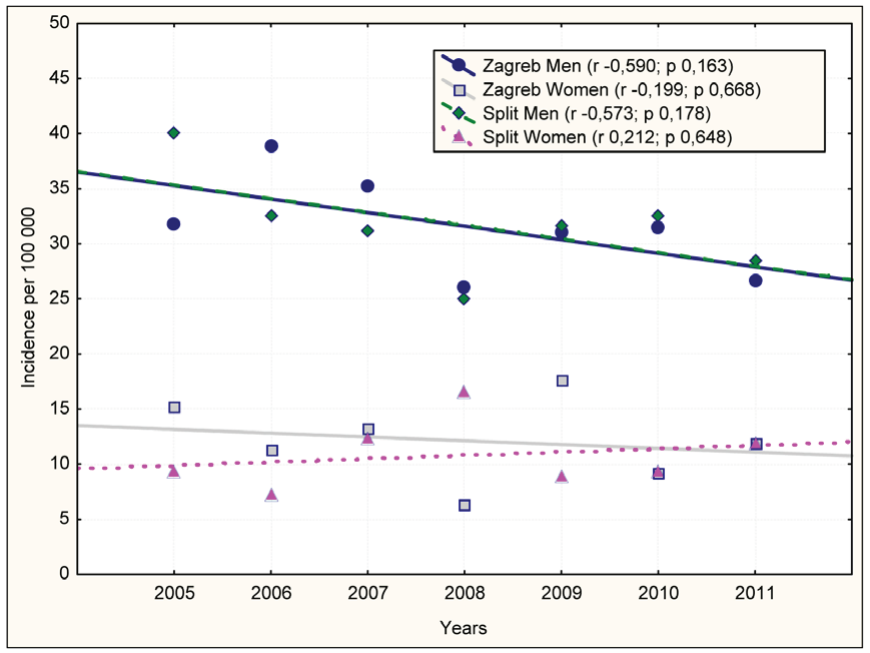
Hospitalization incidence of duodenal ulcer hemorrhage in men and women during the seven- year period (2005-2011) in two regions in Croatia

The majority of patients with APUH were men (66.6%, Table 2). The annual changes of the number of cases with acute gastric and duodenal ulcer hemorrhage for both sexes are shown in Figure 2 and Figure 3, respectively.

**Table 2:** Clinical characteristics of the patients with acute peptic ulcer hemorrhage from Zagreb and Split region

	Gastric ulcer		*P*	Duodenal ulcer		*P*
	Zagreb	Split		Zagreb	Split	
No. patients, n (%)	596 (56.1)	466 (43.9)		467 (40.9)	675 (59.1)	
Age (years), n (%):						
<65	239 (40.1)	164 (35.2)		256 (54.8)	300 (44.5)	
65-80	262 (44.0)	207 (44.4)	0.102	139 (29.8)	250 (37.0)	0.003
>80	95 (15.9)	95 (20.4)		72 (15.4)	125 (18.5)	
Sex, n (%):						
Men	345 (57.9)	299 (64.2)	0.038	327 (70.0)	497 (73.6)	0.181
Women	251 (42.1)	167 (35.8)		140 (30.0)	178 (26.4)	
Clinical presentation, n (%):						
Gastric content						
blood	234 (39.3)	191 (41.0)	0.569	213 (45.6)	302 (46.0)	0.772
coffee ground	362 (60.7)	275 (59.0)		254 (54.3)	373 (54.0)	
Hemodynamic instability	59 (9.2)	12 (2.6)	<0.001	56 (12.0)	4 (0.6)	<0.001
Rockall score						
<3	167 (28.0)	275 (59.0)		174 (37.3)	425 (63.0)	
3-6	358 (60.1)	181 (38.8)	<0.001	252 (53.9)	241 (35.7)	<0.001
≥7	71 (11.9)	10 (2.2)		41 (9.8)	9 (1.3)	
Hemoglobin level (g/L), mean ± standard deviation	93.2 ± 25.2	93.9 ± 24.1	0.706	95.1 ± 24.7	93.6 ± 26.6	0.341
Overall comorbidities, n (%):						
Mild disease (ASA 2)	142 (23.8)	220 (47.2)	<0.001	104 (22.3)	282 (41.8)	<0.001
Moderate/Severe (ASA 3-4)	174 (29.2)	141 (30.3)	0.706	102 (21.8)	167 (24.7)	0.256
*H. pylori* [positive/n (%)]	259/673 (38.5)	95/264 (36.0)	0.478	212/452 (46.9)	275/543(50.6)	0.240
Drugs on presentation, n (%):						
NSAIDs	251 (42.1)	78 (16.7)	<0.001	162 (34.7)	118 (17.5)	<0.001
Aspirin	75 (12.6)	121 (26.0)	<0.001	59 (12.6)	141 (20.9)	<0.001
Antiplatelet drugs	14 (2.4)	0 (0)	0.001	13 (2.8)	0(0)	<0.001
Corticosteroids	4 (0.7)	1 (0.2)	0.076	4 (0.9)	3 (0.4)	0.380
Anticoagulants	30 (5.0)	36 (7.7)	0.072	15 (3.2)	41 (6.1)	0.028
History, n (%):						
Alcohol consumption	155 (26.0)	75 (16.1)	<0.001	134 (28.7)	84 (12.4)	<0.001
Smoking	163 (27.4)	70 (15.0)	<0.001	151 (32.3)	103 (15.3)	<0.001
Previous ulcer disease	168 (28.2)	106 (22.8)	0.044	161 (34.5)	244 (36.2)	0.561
Previous ulcer bleeding	130 (21.8)	87 (18.7)	0.203	126 (27.0)	150 (22.2)	0.065

*Abbreviation: NSAIDs – non-steroidal anti-inflammatory drugs*;* ASA – American Society of Anesthesiologists.

A slight increase in the mean age of patients with APUH was observed in the study period (62.9 to 65.1 years) and the proportion of patients older than 65 years increased from 33.3% in 2005 to 48.7% in 2011. Patients from Zagreb region more often smoked, consumed alcohol, and used NSAIDs. The prevalence of *H. pylori* infection, rebleeding rate, and 30-day case-fatality rate were similar in Zagreb and Split region.

Endoscopic intervention was performed in 977 patients (44.3%), with initial hemostasis achieved in 91.7% of cases (Table 3). Initial hemostasis during endoscopic intervention was achieved more frequently in Zagreb region (94.3% vs 86.6%), as well as mechanical hemostasis with hemoclips (39.2% vs 19.4%). The overall rebleeding rate was 18.2% (187 patients with successful initial endoscopic hemostasis).

**Table 3:** Endoscopic characteristics and clinical outcome of the patients with acute peptic ulcer hemorrhage from Zagreb and Split region

	Gastric ulcer		*P*	Duodenal ulcer		*P*
	Zagreb	Split		Zagreb	Split	
No. patients, n (%)	596 (56.1)	466 (43.9)		467 (40.9)	675 (59.1)	
Signs of hemorrhage, n (%):						
Spurting	32 (5.4)	22 (4.7)	0.607	31 (6.6)	16 (2.4)	<0.001
Oozing	67 (11.3)	45 (9.7)	0.559	76 (16.3)	94 (13.9)	0.350
Visible vessel	148 (25.0)	29 (6.2)	<0.001	79 (16.9)	31 (4.6)	<0.001
Adherent clot	90 (15.2)	65 (14.0)	0.647	88 (18.8)	115 (17.0)	0.385
Pigmented spot	99 (16.7)	47 (10.1)	0.001	66 (14.1)	61 (9.0)	0.008
Clean base ulcer	157 (26.5)	258 (55.4)	<0.001	127 (27.2)	358 (53.0)	<0.001
Ulcer size, n (%):						
<2 cm	500 (84.2)	335 (79.2)	<0.001	420 (90.1)	517 (76.6)	<0.001
≥2 cm	94 (15.8)	131 (28.1)		46 (9.9)	158 (23.4)	
Endoscopic therapy, n (%)	323 (54.2)	156 (33.5)	<0.001	257 (55.3)	241 (35.7)	<0.001
Initial hemostasis, n (%)	320 (95.0)	144 (89.4)	0.02	256 (93.4)	217 (84.8)	0.001
Recurrent hemorrhage, n (%)	44 (7.4)	27 (5.8)	0.304	53 (11.4)	63 (9.3)	0.257
Emergency surgery, n (%)	23 (3.9)	18 (3.9)	0.998	38 (8.1)	21 (3.1)	<0.001
30-day mortality, n (%)	28 (4.7)	28 (6.0)	0.343	21 (4.5)	32 (4.7)	0.847
Blood transfusion (mL), median (range)	780 (270-2540)	740 (140-3460)	0.019	810 (290-4270)	960 (190-5460)	0.006
Hospital stay (days), median (range)	7 (1-45)	7 (1-27)	0.990	7 (1-30)	6 (1-24)	0.342

One hundred patients (4.5%) underwent surgery due to either continuing (12 patients) or recurrent bleeding (83 patients), and perforation (5 patients). More patients were transferred to surgery in Zagreb than in Split region (5.7% vs 3.4%). The majority of patients in whom emergency surgery was performed (59%) had duodenal ulcer located on the posterior wall of the duodenal bulb. Over-sewing of the bleeding vessel (61/100 patients) and partial gastrectomy (37/100 patients) were the most frequent operations.

The overall 30-day case-fatality rate was 4.9%. The rate was similar for patients treated conservatively and those treated surgically (4.5 vs 6.0%), and one-year cumulative mortality rate remained unchanged during the study period. Mortality was higher in patients older than 65 years and in patients with in-hospital hemorrhage recurrence and severe comorbidities. The cause of death in 39.8% patients were severe underlying comorbid illnesses, most common being ischemic heart disease, malignancy, cerebrovascular accidents, and cirrhosis.

## Discussion

Although the introduction of effective antisecretory drugs, alongside *H. pylori* eradication therapies, dramatically improved APUH treatment, the hospitalization incidence of APUH in two regions in Croatia did not change in the study period. In the majority of developed countries, hospitalization rates for APUH are steadily decreasing (11,12), although some studies from England and Finland report an increase (13,14). Concurrently, consumption of aspirin or NSAIDs is increasing, particularly among the elderly patients (15). This is consistent with our data demonstrating a significant increase in the proportion of elderly patients with peptic ulcer bleeding, with a high proportion of patients who were taking NSAIDs. Our study found that the hospitalization for peptic ulcer hemorrhage per 100 000 remained unchanged, which is similar to Danish and Dutch studies (16,17).

In Western countries in the second half of the 20th century peptic ulcer was no longer a disease predominant in men but had a comparable prevalence in both sexes (18). In our population, greater prevalence was observed in men.

There appears to be a considerable variation in the rates and pattern of peptic ulcer among different regions in the world, but there is a limited number of population-based studies to support these conclusions (19). Zagreb is the largest city in Croatia situated in the northern part of the country, with predominantly continental European climate and typical central-European cuisine, whereas the Split is the largest city in the southern part of the country with Mediterranean climate and cuisine. These two cities are fairly good representatives of two different life styles. One-year cumulative hospitalization incidence for peptic ulcer bleeding was significantly higher in the northern part of Croatia. This finding can be explained by greater NSAID and tobacco consumption in northern Croatia, with no difference in *H. pylori* infection rates between the regions. The fact that the majority of APUH patients from northern Croatia had gastric ulcers additionally supports the role of these etiologic factors. Duodenal ulcers were more frequent in the southern part of Croatia. Similar north-south differences in China and India have been attributed to regional dietary habits (20,21).

Along with the significant increase in the proportion of elderly patients presenting with APUH, we observed that up to 52.2% of our patients had been exposed to drugs potentially harmful to the upper GI tract (NSAIDs, aspirin, antiplatelet agents, corticosteroids, and anticoagulants). At the same time, low percentage of gastroprotective drugs usage (up to 10%) was found in all age groups. This fact stresses the need for prescription of gastroprotective medications in elderly patients at high risk of upper GI bleeding.

The factors considerably affecting the rebleeding rate were hemodynamic instability upon admission, moderate to severe comorbidities, and the presence of duodenal ulcer larger than 2 cm in diameter with a spurting bleeding (5). Causes of mortality after APUH are more often related to cardiac or multiorgan failure than to the bleeding itself, particularly in older and co-morbid patients (22,23). The 30-day case-fatality in our patients with APUH was 4.9%, which is in accordance with other similar studies (24-28).

In conclusion, the hospitalization incidence of APUH in two populations in Croatia did not change over a seven-year period and it was significantly higher in Zagreb region. The incidence of acute duodenal ulcer hemorrhage remained unchanged, whereas the incidence of acute gastric ulcer hemorrhage increased. The results of this study allow us to monitor epidemiological indicators of APUH and compare data with other countries.
